# Unaltered soil microbial community composition, but decreased metabolic activity in a semiarid grassland after two years of passive experimental warming

**DOI:** 10.1002/ece3.6862

**Published:** 2020-10-08

**Authors:** Chao Fang, Wenbin Ke, Matteo Campioli, Jiuying Pei, Ziqiang Yuan, Xin Song, Jian‐Sheng Ye, Fengmin Li, Ivan A. Janssens

**Affiliations:** ^1^ Institute of Ecology School of Applied Meteorology Nanjing University of Information Science and Technology Nanjing China; ^2^ State Key Laboratory of Grassland Agro‐ecosystems Institute of Arid Agroecology School of Life Sciences Lanzhou University Lanzhou China; ^3^ PLECO (Plants and Ecosystems) Department of Biology University of Antwerp Wilrijk Belgium; ^4^ State Key Laboratory of Frozen Soil Engineering Northwest Institute of Eco‐Environment and Resources Chinese Academy of Science Lanzhou China

**Keywords:** climate change, heterotrophic respiration, microbes, nutrient availability, plant production

## Abstract

Soil microbial communities regulate soil carbon feedbacks to climate warming through microbial respiration (i.e., metabolic rate). A thorough understanding of the responses of composition, biomass, and metabolic rate of soil microbial community to warming is crucial to predict soil carbon stocks in a future warmer climate. Therefore, we conducted a field manipulative experiment in a semiarid grassland on the Loess Plateau of China to evaluate the responses of the soil microbial community to increased temperature from April 2015 to December 2017. Soil temperature was 2.0°C higher relative to the ambient when open‐top chambers (OTCs) were used. Warming did not affect microbial biomass or the composition of microbial functional groups. However, warming significantly decreased microbial respiration, directly resulting from soil pH decrease driven by the comediation of aboveground biomass increase, inorganic nitrogen increase, and moisture decrease. These findings highlight that the soil microbial community structure of semiarid grasslands resisted the short‐term warming by 2°C, although its metabolic rate declined.

## Graphical abstract

This study showed that there were no warming effects on soil microbial community composition and on soil microbial biomass, warming decreased soil microbial metabolic rate, and soil microbial community structure resisted to short‐term warming by 2°C.

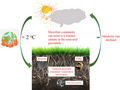

## 1 INTRODUCTION

The Earth's surface temperature has increased by approximately 1.0°C since the industrial revolution, and is projected to further increase until the end of this century (Allen et al., 2018). Ecosystem carbon sequestration is slowing down, which has been attributed to a potential shift from a fertilization‐dominated period due to the effects of CO_2_ and nitrogen fertilization to a warming‐dominated period (Penuelas et al., 2017). It has been suggested that a one‐degree increase in temperature could induce a loss of 30 Pg soil carbon (C) year^−1^ equivalent to about 2–3 times as the amount emitted annually by human‐related activities (Crowther et al., 2016; Penuelas et al., 2017). Many studies have focused on understanding how increased temperature influences the CO_2_ efflux from soil mainly through soil organic matter (SOM) decomposition (Davidson & Janssens, 2006; Fernández‐Martínez et al., 2018; Melillo et al., 2017; Pries et al., 2017; Zong et al., 2018). Even though soil microbial community is the engine responsible for this SOM decomposition (Ali et al., 2018; Cheng et al., 2017; Frostegård & Bååth, 1996; Zhou et al., 2016), a general conclusion as to how soil microbial community composition will be affected by warming remains elusive (Radujkovic et al., 2018). Therefore, knowledge about the warming responses of microbial communities is still needed to better understand global C cycling in the future warmer climate.

Profiles of phospholipid fatty acids (PLFAs) have been widely used to investigate microbial biomass and community composition because PLFAs only remain intact in active or dormant cells and hence are characteristic biomarkers for living microorganisms (Evershed et al., 2006; Feng & Simpson, 2009; Frostegård & Bååth, 1996; Wei et al., 2014; Xu et al., 2015). Previous studies have shown that warming responses of total soil microbial community size (i.e., total PLFAs) are highly variable and complex. For example, warming was found to have a negative (Ali et al., 2018; Xue et al., 2016), no (Schindlbacher et al., 2011), or even a positive (Wang et al., 2017; Zhang, et al., 2015) effect on total PLFAs. These different warming responses of total soil microbial community size may result directly from shifts of microbial functional groups or indirectly from changes in soil and plant properties. For example, the warming treatment directly led to rapid shifts in the structure of the soil microbial community with significantly increased abundance of actinomycete biomarkers and decreased abundance of fungi (Xiong et al., 2016). Warming may alter soil pH, soil resource availability, plant community composition, and plant production (Bai et al., 2019; Fang et al., 2018; Guan et al., 2018; Li et al., 2017; Xu et al., 2013; Zi et al., 2018), which can all affect the soil microbial community (Feng & Simpson, 2009; C. Wang et al., 2017; Xiong et al., 2016; Xue et al., 2016). For example, soil pH positively correlated with fungi:bacteria ratio (Xiong et al., 2016). Decreases in available nutrient sources lead to faster decline in fungi and gram‐negative bacteria than gram‐positive bacteria (Feng & Simpson, 2009). Faster root turnover could increase bacterial and fungal PLFAs (Wang et al., 2017). Therefore, the change in microbial community in response to warming may result from its coupling with multiple environmental factors. However, the mechanisms underlying warming responses of the microbial community and its different functional groups still remain unclear (Li et al., 2017; Pendall, 2018).

The overall effect of warming on soil C pool can be inferred by assessing soil respiration rate (Liski et al., 1999; Luo et al., 2009; Walker et al., 2018), which is crucially affected by microbial metabolic rate (i.e., microbial respiration rate; Fang et al., 2018; Hicks Pries et al., 2015; Kuzyakov, 2002; Xuhui Zhou et al., 2007). Studies over the past decades have shown that the warming response of microbial respiration is highly variable. Previous studies showed that warming could stimulate microbial respiration due to increased microbial biomass C (Liu et al., 2019), soil nutrient availability (Ali et al., 2018), root exudates (Li et al., 2013), enzymatic activity (Bragazza et al., 2012; Li et al., 2017), or plant production (Euskirchen et al., 2009). Some other studies showed opposite results; that is, microbial respiration inhibited by warming, which resulted from decreased soil water availability (Fang et al., 2018; Liu et al., 2009), labile C (Li et al., 2019), microbial biomass C (Chen, et al., 2016), or enzymatic activity (Garcia‐Palacios et al., 2018). As a result, these contrasting responses of microbial metabolic rate to warming led to large uncertainties and contradictory predictions of climate‐C feedbacks (Bradford et al., 2010; Hartley et al., 2007, 2009). Thus, it is crucial to clarify the warming responses of microbial metabolic rate in terrestrial ecosystems, especially in semiarid ecosystems, where low soil water availability is more likely to induce unexpected warming responses (Song et al., 2019).

The Loess Plateau of Northwestern China, having an altitude range of 1000–1600 m and covering an area of 640 000 km^2^, is one of the most eroded regions in the world (Chen et al., 2007; Turner et al., 2011). It has been projected that the ecosystems in eroded or degraded regions with high altitude are sensitive to climate change (Fan & Wang, 2011; Thomas et al., 2004; Wang et al., 2012). Loess Plateau will experience a warmer climate with a warming rate of 0.113–0.558°C/10 year during 2020–2100 under four RCP scenarios (Peng et al., 2017). With long‐term warming in the future, some stations in the Loess Plateau will experience a higher frequency of drought based on two regional climate models (Sun et al., 2019). Drought could limit plant growth (Walter et al., 2011), potentially decreasing ecosystem carbon uptake (Berdugo et al., 2020). Thus, the Loess Plateau plays an important role in regulating global C cycle and climate change (Shi et al., 2011; Ueyama et al., 2009). Grassland is the most widely distributed vegetation type in the region, which accounts for about 42% of the total land area (Li, Li, & Lü, 2016). Zheng et al. (2019) showed climate change contributed more compared with human activities to the changes in NDVI in the Loess Plateau based on MODIS and net primary productivity model. The semiarid area, accounting for 60% of the Loess Plateau, is characterized by low precipitation, low soil water content, and low vegetation cover, as well as severe soil erosion (Chen et al., 2007; Gao et al., 2009; Ye et al., 2013; Zhang, 1989). Severe soil erosion could cause land‐quality decline and ecosystem production reduction (Chen et al., 2007). Thus, the grassland ecosystems in the semiarid region of Loess Plateau are extremely vulnerable to climate change. Previous studies have shown that warming may decrease soil water availability and plant cover in semiarid grasslands of the Loess Plateau (Fang et al., 2017, 2018), and potentially decrease microbial metabolic rate. Waldrop and Firestone (2006) showed that increased water availability significantly stimulated bacterial growth, while exerting no profound impacts on soil fungi. Some other studies showed significant increases in both bacterial and fungal PLFAs with increasing soil water availability in dryland ecosystems (Bi et al., 2012; Clark et al., 2009). More drought‐tolerant microorganisms such as fungi and actinobacteria usually benefit from dry conditions. Therefore, decrease in soil moisture might lead to an increase in fungi/bacteria ratio in semiarid regions. To evaluate the effects of warming on soil microbial dynamics, we conducted a manipulative experiment in a typical semiarid grassland on the Loess Plateau, China. We hypothesized that the changes in soil environmental characteristics due to warming will (a) decrease microbial metabolic rate and soil microbial biomass; and (b) shift microbial community composition toward higher fungi:bacteria ratio.

## 2 MATERIALS AND METHODS

### 2.1 Study area

The experiment was conducted from April 2015 to December 2017 in a fenced grassland at the Semiarid Ecosystem Research Station (Lanzhou University) on the Loess Plateau of China (36°02′N, 104°25′E). The area has a medium‐temperate semiarid climate. The altitude of the site is 2,400 m above sea level. According to the meteorological record from 1955 to 2013, mean annual air temperature is 6.5°C, and mean annual precipitation is 305 mm, with 80% of the annual rainfall occurring during the growing season (April–October). The mean annual pan evaporation is roughly 1,300 mm. The soil is clarified as Heima soil (Calcic Kastanozem, FAO Taxonomy), with a high percentage of silt (around 76%). The study area was sown with *Melilotus suaveolens L*. (a biennial herb) in April 2003 to facilitate the revegetation of degraded land for improving the vegetation cover and reduce soil erosion. Then, the *Melilotus suaveolens L*. grassland was fenced and unmanaged. The main vegetation type at the study site was dominated by *Heteropappus altaicus Novopokr*., *Stipa breviflora Griseb*., and *Artemisia capillaris* through secondary succession.

### 2.2 Experiment design

**Figure 1:**
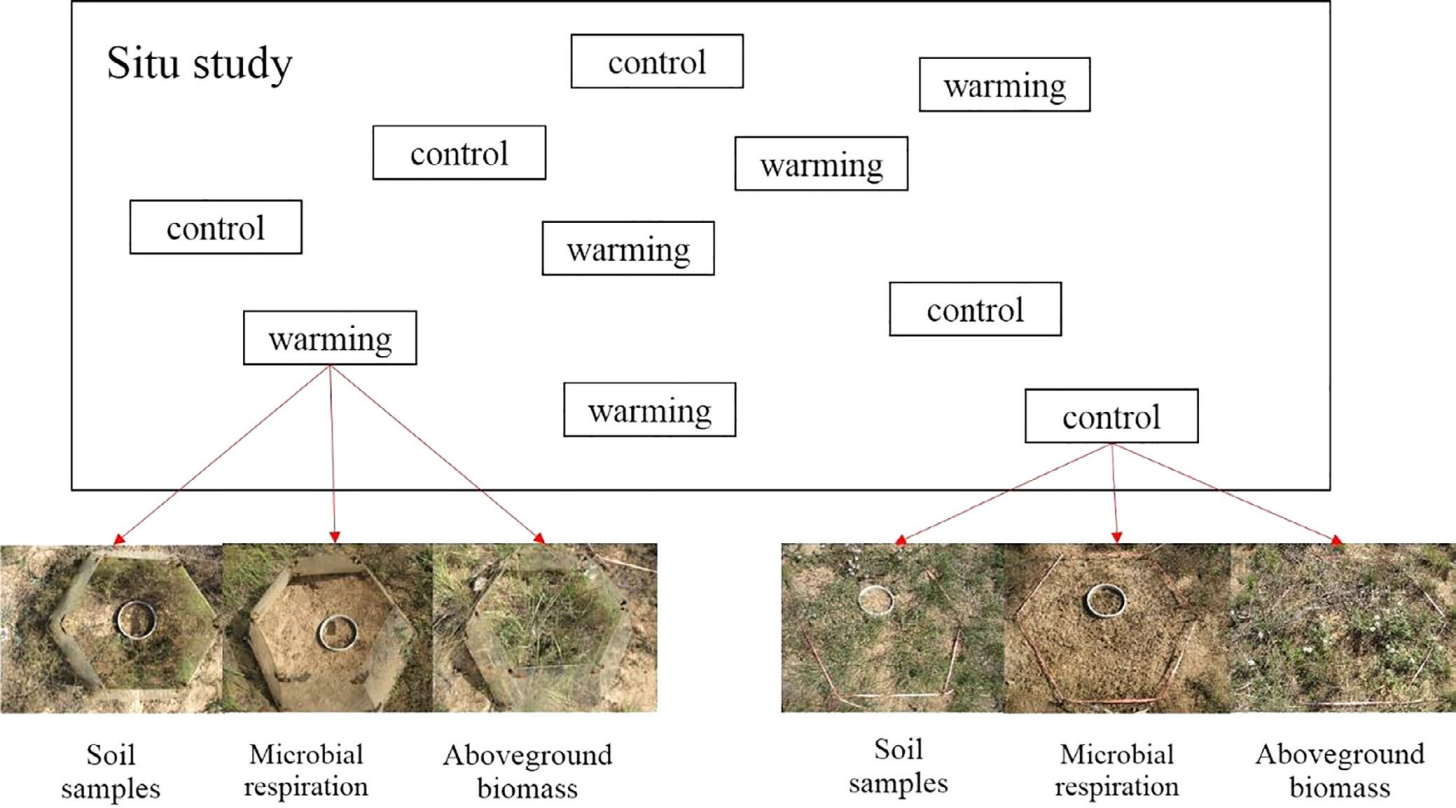
Layout of the experiment design. In each plot, three subplots were set to measure soil properties, microbial respiration, and aboveground biomass, respectively

Two treatments in this study were included: control and warming (Figure 1). The open‐top chambers (OTCs) were used as passive warming devices, which were shown to be effective in a large number of experiments. The OTC was a hexagonal design with sloping sides of 40 cm × 50 cm × 32 cm; the OTCs were placed on the soil surface and provided year‐round warming to the enclosure. For each treatment, three randomly chosen subplots with a regular hexagon (0.5 m sides for a total of 0.65 m^−2^) were selected for soil sampling, microbial respiration, and aboveground biomass measurements, respectively (Figure 1). In warming treatment, one OTC was used in each subplot, that is, total three OTCs for three subplots (Figure 1). Microtrenching method was adopted in October 2014 for soil microbial respiration measurement within each subplot (see below). The trenches (0.1 m wide and 0.5 m deep) were excavated and then lined with nylon mesh (0.038 mm mesh size) to prevent root growth into the trenched area, yet allowing the movement of water and soil nutrients (Zhang et al., 2014). The trenches were then refilled with the same soil. The area inside the trench was then kept vegetation‐free by cutting those regrowing plant manually throughout the study period. The measurement method applied here for respiration has been widely used in manipulative in situ experiments (Fang et al., 2017; Hanson et al., 2000; Kuzyakov et al., 2002; Liu et al., 2016; Moyano et al., 2007; Zhang et al., 2015). It is true that this method, that is, trenching method, may cause biases in estimating microbial respiration including underestimations (due to exclusion of roots; Hanson et al., 2000; Ngao et al., 2007) and overestimations (due to higher soil moisture; Yan et al., 2009). Nevertheless, Zhang et al. (2014) suggested that the trenching method is a feasible way to measure soil microbial respiration as the soil structure is not disturbed, and the transfers of soil nutrient and water resources are allowed between inside and outside of the trenched plots. More details about the measurement method, and its advantages and disadvantages are reported in Fang et al. (2018).

### 2.3 Soil sampling

In August 2017, soil samples were collected using a soil auger with an inner diameter of 2 cm. Five soil cores in each plot were taken to a depth of 20 cm and mixed into one soil sample. Soil samples were placed on ice and immediately transported to the laboratory. Afterward, each soil sample was divided into three parts. One part was stored at 4°C for soil moisture, microbial biomass (C and N), and inorganic nitrogen (N) measurements. Another part was stored at −20°C for PLFA measurement. The third part was air‐dried for soil pH, organic carbon (SOC), light (LOC) and heavy (HOC) fractions of SOC, total *N* (TN), and phosphorus (P) measurements.

### 2.4 Soil physicochemical properties and PLFAs

Soil water content was determined gravimetrically after oven‐drying at 105°C. Soil pH was measured in a soil–water solution ratio of 1:2.5 (w/w) using a glass electrode. Soil total organic carbon was measured using the Walkley and Black method (Nelson & Sommers, 1982). The density fractions of light and heavy fractions of soil organic carbon were first extracted using the method from Gregorich and Ellert (1993), and then determined by the Walkley and Black method (Nelson & Sommers, 1982). Soil total N was measured using the Kjeldahl digestion method (Bremner & Mulvaney, 1982). Soil total phosphorus was determined by the molybdate colorimetric method (O'Halloran & Cade‐Menun, 2006). Soil available P was determined with the Olsen method (Olsen et al., 1954). Soil inorganic N was determined by San^++^ Automated Wet Chemistry Analyzer (Skalar, Breda, the Netherlands) after extraction with KCl (2 mol/L) (Miller & Keeney, 1982). Soil microbial biomass C and N were determined by chloroform–fumigation–extraction method (Brooks et al., 1985; Vance et al., 1987).

PLFAs were extracted and quantified using a modified method by Bossio and Scow (1998). Briefly, water content of the soil samples was measured prior to the procedure for adjusting the method and for further calculations. Lipids were extracted with a chloroform: methanol: phosphate buffer mixture (1:2:0.8 v/v/v, pH 4.0) from 8 g frozen soil. Throughout the procedure, teflon tubes and caps were hexane‐rinsed, and all glassware was heated to 121°C for 3 hr to sterilize and remove exogenous lipids. The phospholipids were separated from neutral lipids and glycolipids on a silica acid column (0.5 g Si, Supelco, Inc., Bellefonte, Penn). Polar lipids were eluted and then subjected to a mild alkaline methanolysis after the addition of an internal standard—methyl nonadecanoate fatty acid (19:0). The resulting fatty acid methyl esters (FAMEs) were separated, quantified, and identified with an Agilent 6,890 gas chromatographer (GC; Agilent Technologies, Palo Alto, CA, USA) equipped with a 19091B‐102 flame ionization detector (Agilent Technologies). Samples were injected in split‐less mode (injector temperature: 230°C) and separated using a DB23 column (60 m × 0.25 mm × 0.25 μm; Agilent, Vienna, Austria) with 1.5 ml/min helium as the carrier gas. GC operating conditions were as follows: 1.5 min at 70°C, 30°C min^−1^ to 150°C; 1 min at 150°C, 4°C min^−1^ to 230°C; and 15 min at 230°C; N_2_ was used as the make‐up gas, and air was used to support the flame. The fatty acid methyl esters of these samples were identified based on chromatographic retention time according to the MIDI Sherlock Microbial Identification System in the standard EUKARY chromatographic program (Microbial ID, Inc., Newark, DE, USA). The concentrations of PLFAs were standardized by the reference concentrations of internal standard (19:0) at a retention time of 71.14 min.

The terminal‐branched saturated PLFAs a13:0, i13:0, a15:0, i15:0, a16:0, i16:0, a17:0, and i17:0 were used as markers for gram‐positive bacteria. The mono‐unsaturated and cyclopropyl saturated PLFAs 2OH 16:1, 16:1ω7c, cy17:0, and cy19:0 were used as markers for gram‐negative bacteria. The bacterial markers were 12:0, 14:0, 16:0, 17:0, 18:0, G+, and G−. The 18:2ω6, 9c was used as a fungal PLFA marker. The methylic, midchain branched, saturated PLFA peaks 10 Me 16:0, 10 Me 17:0, and 10 Me 18:0 were used as indicators for actinomycetes. The PLFAs 16:1ω5c and 16:1ω11 represented arbuscular mycorrhizal fungi. Total PLFA concentration was used as an index of the total microbial biomass. The sum of PLFAs characteristic of total bacteria (Tbacteria), gram‐positive bacteria (G+), gram‐negative bacteria (G−), actinomycetes (ACT), fungi (F), and arbuscular mycorrhizal fungi (AMF) was used to determine broad taxonomic microbial groupings. We characterized microbial C/N, F/B, G+/G−, and ratio of the sum of cyclopropyl PLFAs to the sum of their monoenoic precursors (cy 17:0 + cy19:0)/(16:1ω7 + 18:1ω7) (CM) as the indicators of physiological or nutritional stress in microbial communities (Bossio & Scow, 1998; Fanin et al., 2019; Moore‐Kucera & Dick, 2008; Schindlbacher et al., 2011).

### 2.5 Microbial respiration

Soil microbial respiration was measured from April 2015 until December 2017. A polyvinyl chloride (PVC) collar (11 cm in diameter and 8 cm in height) was inserted into the soil to a depth of 5 cm at the center of each trenched area for the duration of the study, and a Li‐8100 infrared gas analyzer (LI‐COR Biosciences, Lincoln, Nebraska, USA) was used to measure the soil CO_2_ efflux in the collar. Microbial respiration was measured twice every month in the growing season (April to October) and once per month outside of growing season. All microbial respiration measurements (2 treatments with 5 replicates) were carried out between 09:00 and 11:00 a.m. (local time). To ensure the smallest fluctuation of CO_2_ concentration in the Li‐8100 chamber, each measurement took roughly 2–3 min.

### 2.6 Aboveground biomass

We used a nondestructive sampling method to estimate aboveground biomass (AGB) on 13 August 2016. The height of each species was measured in each plot, and AGB was calculated using the allometric relationship between biomass and height for each species (Fang et al., 2018).

### 2.7 Air temperature, soil temperature, and moisture

One warming plot and one control plot were randomly selected for continuous measurements of air temperature, soil temperature, and soil moisture. Air temperature was recorded by using HOBO^®^ T/RH U23‐002 Data Loggers (Onset Computer Corporation, Bourne, MA, USA); soil temperature and volumetric soil moisture were recorded at a depth of 5 cm using a Watchdog 1000 Series Micro station‐T/RH (Spectrum Technologies Inc., Plainfield, IL, USA). All data were recorded at a 1‐hr interval. Although soil temperature and moisture were recorded only in one plot of each warming and control treatments, the values recorded were consistent with the ones conducted in a parallel nearby field (Fang et al., 2018). Warming increased air and soil temperature by 1.7 ± 0.3°C and 2.0 ± 0.4°C, respectively, whereas it decreased soil volumetric water by 1.7 ± 0.74% and 1.8 ± 1.3% in nontrenched and trenched plot, respectively (Fang et al., 2018). More details about the effects of warming on air or soil temperature and moisture were described in Fang et al. (2018).

### 2.8 Statistical analysis

We performed a paired *t* test to compare the difference in soil temperature and moisture for the paired warming and control treatments. Independent‐samples *t* test was used to verify the effect of soil warming on SWC, pH, SOC, TN, TP, AP, C/N, inorganic N, LOC, HOC, aboveground biomass, PLFAs, G+/G−, F/B, MC, microbial respiration, MBC, MBN, MBC/MBN, and microbial metabolic quotient. Significant differences were evaluated at the level *p* < .05. Pearson's correlation analysis was used to evaluate the correlations among soil microbial community, indicators of environment stress, AGB, pH, and soil nutrients. Structural equation modeling (SEM) was performed using AMOS 21.0 to quantify the relative importance of the potential direct and indirect pathways in mediating the soil warming effects on soil microbial respiration based on conceptual modeling (Figure S1), significance of the regression equation (Pearson's correlation analysis; Table 1), the goodness of model fit, and logical reasoning. As the soil samples were taken in August 2017, mean value of soil microbial respiration from June to September in 2017 was used in the SEM for more accurately evaluating the driving mechanisms of warming on microbial respiration. All statistical analyses were performed using SPSS 21.0 (SPSS Inc., Chicago, IL, USA).

**Table 1:** Pearson's correlation coefficients (r values) between soil microbial community and soil nutrients and indicators of environment stress

Microbes	AGB	pH	SWC	ION	SOC	LOC	HOC	TN	TP	AP	C/*N*	MBC/MBN	G+/G−	F/B	CM
TPLFAs	0.36	0.17	0.25	0.11	0.43	0.08	−0.43	0.03	−0.66*	−0.29	0.46	0.06	−0.65*	0.84**	−0.72*
Actinomycetes	0.57	0.09	0.02	0.16	0.38	0.23	−0.28	0.07	−0.47	−0.12	0.37	−0.01	−0.67*	0.80**	−0.59*
Tbacteria	0.54	0.06	0.12	0.23	0.32	0.26	−0.38	0.11	−0.63*	−0.23	0.28	−0.03	−0.75*	0.88**	−0.68
G+	0.55	0.10	0.15	0.20	0.40	0.21	−0.40	0.09	−0.60*	−0.20	0.39	−0.01	−0.67*	0.84^**^	−0.68*
G−	0.56	0.01	0.09	0.29	0.27	0.32	−0.38	0.14	−0.65*	−0.20	0.21	−0.02	−0.78**	0.89^**^	−0.68*
Fungi	0.52	0.06	0.16	0.24	0.27	0.29	−0.41	0.10	−0.66*	−0.24	0.24	−0.12	−0.74*	0.91^**^	−0.73*
AMF	0.55	−0.07	0.21	0.40	0.26	0.43	−0.44	0.26	−0.73*	−0.16	0.12	0.05	−0.67*	0.85^**^	−0.75*
MBC	0.56	−0.29	−0.42	0.34	0.13	−0.13	−0.10	−0.17	−0.34	−0.20	0.28	−0.55	−0.75*	0.43	−0.01
MBN	0.48	−0.14	0	0.25	−0.04	−0.17	−0.30	0	−0.37	−0.22	−0.02	−0.33	−0.52	0.37	−0.03
Rh	−0.56	0.79^**^	0.75^**^	−0.74*	0.21	−0.24	−0.06	0.16	0.02	−0.47	−0.11	−0.15	0.13	0.22	−0.36
C/*N*	0.29	0.28	−0.07	−0.15	0.79	−0.16	−0.01	−0.20	0.04	−0.21	1.00	0.28	0.04	0.02	0.05
MBC/MBN	−0.02	−0.28	−0.62	0.15	0.03	0.21	0.29	−0.37	0.05	0.04	0.28	1.00	−0.19	0.01	−0.01
G+/G−	−0.42	0.24	0.23	−0.38	0.07	−0.2	0.25	0.05	0.65*	0.27	0.04	−0.19	1.00	−0.8	0.44
F/B	0.32	0.02	0.2	0.23	−0.10	0.16	−0.62	−0.16	−0.82*	−0.20	0.02	0.01	−0.80**	1.00	−0.84**
CM	−0.10	0.03	−0.41	−0.26	0.12	−0.23	0.65*	0.11	0.82*	0.10	0.05	−0.01	0.44	−0.84**	1.00

Note

AGB, aboveground biomass; AMF, arbuscular mycorrhizal fungi; AP, soil availability phosphorus; C/*N*, soil organic carbon/total nitrogen; CM, (cy 17:0 + cy19:0)/(16:1ω7 + 18:1ω7); F/B, fungi, bacteria; G−, gram‐negative bacteria; G+, gram‐positive bacteria; HOC, heavy organic carbon; ION, inorganic nitrogen; LOC, light organic carbon; MBC/MBN, soil microbial biomass C/soil microbial biomass *N*; MBC, soil microbial biomass C; MBN, soil microbial biomass *N*; pH, soil pH; Rh, microbial respiration; SOC, soil organic carbon; SWC, soil gravitational water content; Tbacteria, total bacteria; TN, soil total nitrogen; TP, soil total phosphorus; TPLFAs, total PLFAs. *p < .05; **p < .01.

## 3 RESULTS

### 3.1 Soil water and chemical properties

**Figure 2:**
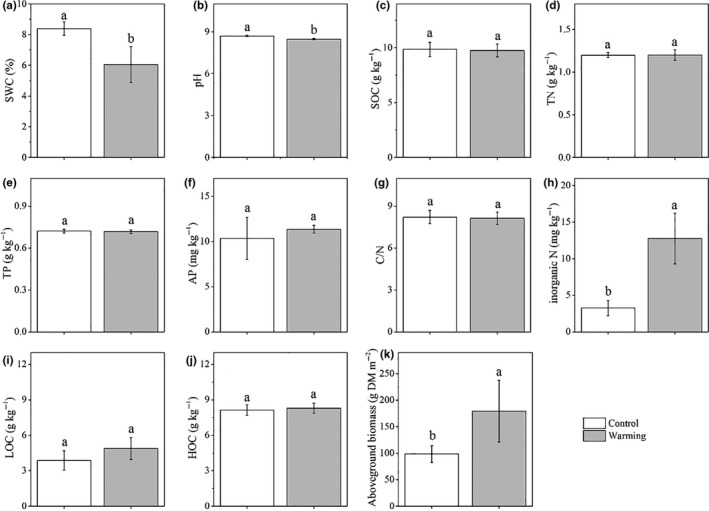
Soil chemical characteristics at a soil depth of 20 cm. SWC, soil gravitational water content (a); pH, soil pH (b); SOC, soil organic carbon (c); TN, soil total nitrogen (d); TP, soil total phosphorus (e); AP, soil availability phosphorus (f); C/N, soil organic carbon/total nitrogen (g); inorganic N, inorganic nitrogen (h); LOC, light organic carbon (i); HOC, heavy organic carbon (j); and AGB, aboveground biomass (k). Different letters represent significant difference at *p* < .05. Data shown are means ± standard deviation (*n* = 5)

Warming significantly decreased SWC from 8.38 ± 0.43% to 6.05 ± 1.18% (Figure 2a). Soil pH was significantly lower in the warmed treatment than in the control treatment (Figure 2b). Inorganic N and AGB were significantly higher in the warmed treatment than in the control treatment (Figure 2h,k). There were no significant differences in SOC, TN, TP, AP, C/N, LOC, and HOC between the warmed and control treatments (Figure 2c–g and i,j).

### 3.2 Soil microbial properties

**Figure 3:**
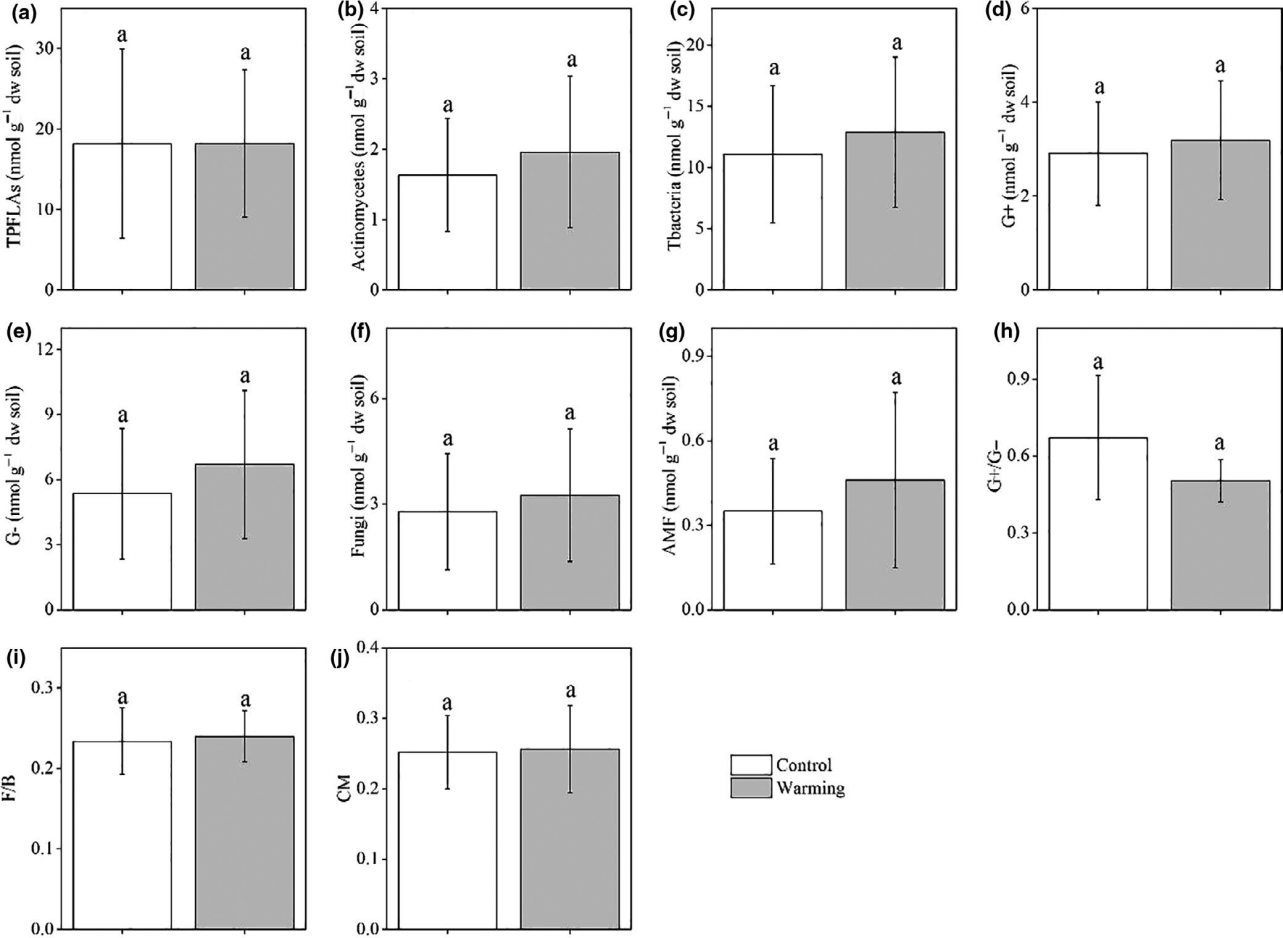
PLFAs of total microbial community (a), actinomycetes (b), Tbacteria (c), G+ (d), G− (e), fungi (f), AMF (g), and G+/G− (h), F/B (i), and CM (j). TPLFAs, total PLFAs; Tbacteria, total bacteria; G+, gram‐positive bacteria; G−, gram‐negative bacteria; AMF, arbuscular mycorrhizal fungi; F, fungi; B, bacteria; CM, (cy 17:0 + cy19:0)/(16:1ω7 + 18:1ω7). Different letters represent significant difference at *p* < .05. Data shown are means ± standard deviation (*n* = 5)

**Figure 4:**
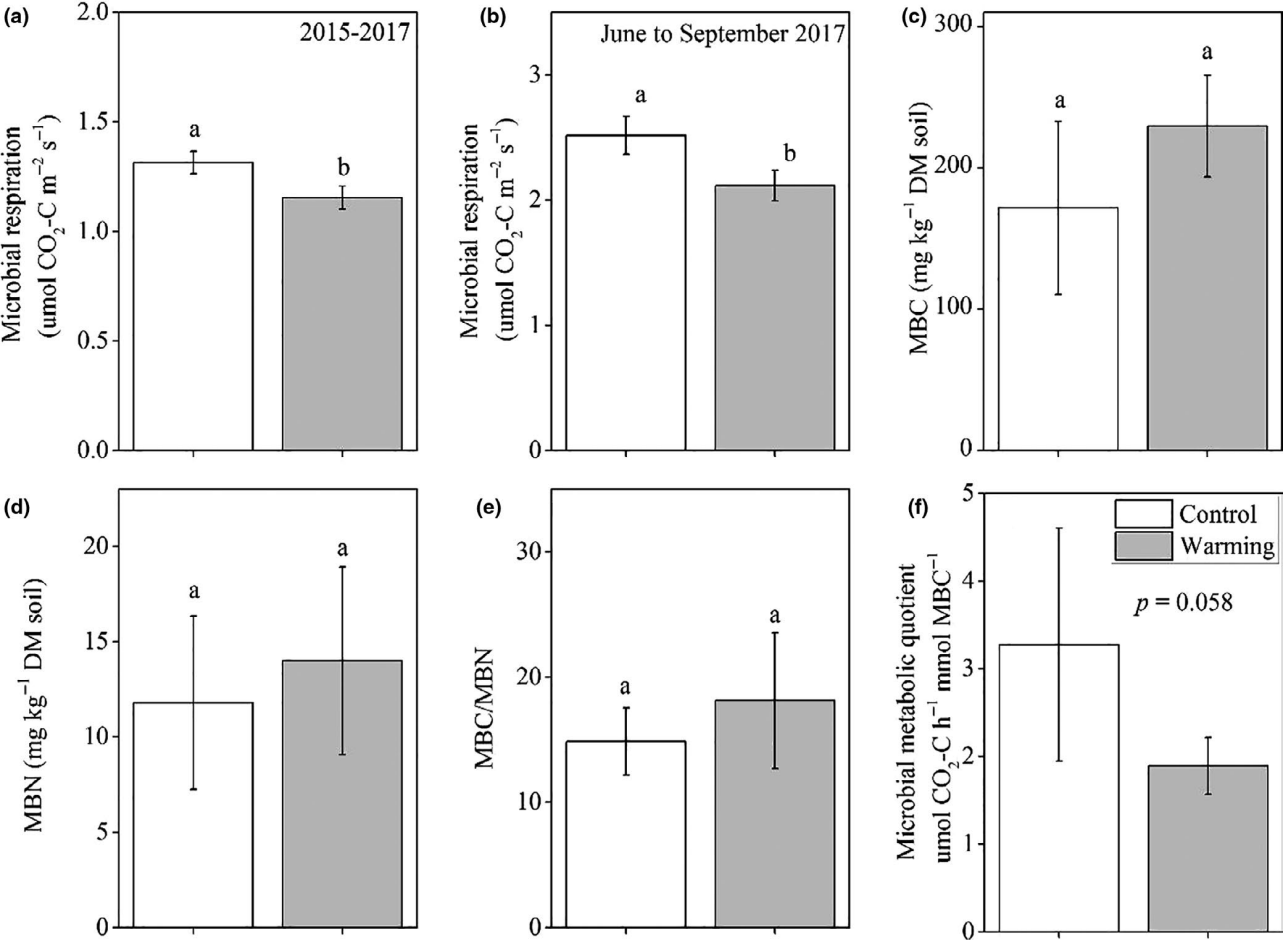
Soil microbial respiration rate and biomass. Mean value of microbial respiration during April 2015–December 2017 (a), mean value of microbial respiration during June–September 2017 (b), soil microbial biomass C (MBC) (c), *N* (MBN) (d), MBC/MBN (e), and microbial metabolic quotient (f) at the 0–20 cm soil depth. Different letters represent significant difference at *p* < .05. Data shown are means ± standard deviation (*n* = 5)

The PLFAs of bacteria and fungi accounted for 67.4% and 15.4% of TPLFAs in the control treatment, respectively. There were no significant differences in TPLFAs, actinomycetes, Tbacteria, G+, G−, fungi, AMF, G+/G−, F/B, and CM between the warmed and control treatments (Figure 3). Microbial respiration was significantly lower in the warmed treatment than in the control treatment (Figure 4a,b). No significant differences in MBC, MBN, and MBC/MBN were observed between warming and control treatments (Figure 4c–e). Microbial metabolic quotient was lower in warming plots than in control plots (*p* = .058, Figure 4f).

### 3.3 Relationships of soil microbial properties with soil chemical properties

**Figure 5:**
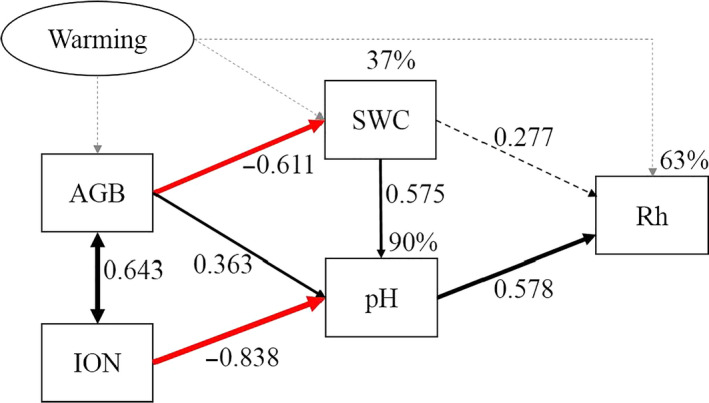
Structure equation modeling (SEM) with variables (boxes) and potential causal relationships (arrows) for soil microbial respiration (χ^2^ = 5.154, *df* = 3, *p* = .161 > 0.05, CFI = 0.937 > 0.9). Rh, microbial respiration; SWC, soil water content; pH, soil pH value; AGB, aboveground biomass; and ION, inorganic nitrogen (*n* = 10). Single‐headed arrows represent the hypothesized direction of causation. Numbers next to single‐headed arrows are standardized path coefficients, which indicate the effect size of the relationship. The double‐headed arrow represents the covariance between related variables. Red and black arrows indicate negative and positive relationship. The proportion of variance explained appears above each response variables in the model. Solid line means significance, and dash line means nonsignificance. Due to insufficient temperature data (only continuous measurements in two plots in two treatments) for a SEM analysis, the gray dashed lines represent the conceptual warming effect

TPLFAs, actinomycetes, Tbacteria, G+, G−, fungi, and AMF were significantly correlated with TP, G+/G−, F/B, and CM, except for the absence of a relationship between actinomycetes and TP (Table 1). Significant relationships were observed between G+/G− and F/B, and G+/G and CM on the one hand, and F/B and CM on the other hand (Table 1). Microbial respiration was significantly positively correlated with pH and SWC, and negatively correlated with inorganic N (Table 1). The final SEM of microbial respiration showed that AGB, inorganic N, SWC, and pH explained 63% of the variance in microbial respiration (Figure 5). Taking the direct and indirect effects together, inorganic N was the most important predictor shaping the variance of microbial respiration (Figure 5). Specifically, AGB, inorganic N, and SWC affected microbial respiration through directly regulating pH (Figure 5).

## 4 DISCUSSION

Our hypothesis that increased temperature would decrease soil microbial biomass and alter community composition was not supported, results showed that two years of warming did not affect soil microbial biomass and community composition. This finding is consistent with that observed in the forest soils with soil warming of 4–5°C (Contosta et al., 2015; Schindlbacher et al., 2011) and an alpine meadow with soil warming of 0.44°C (Zi et al., 2018).

The mechanism underlying the lack of a warming response in microbial biomass and community composition might result from the abiotic and biotic factors coupled in this study. In our study, warming significantly increased soil temperature and stimulated aboveground biomass, which resulted in increased litter production, and this accelerated soil N cycling (e.g., increased N availability) (Figure 2), providing a favorable environment for the soil microbial community. In a laboratory incubation study, increased temperature decreased total PLFAs due to decreased substrate availability (Ali et al., 2018). In one other laboratory soil warming study, Zhou et al. (2017) reported that increased total PLFAs at higher temperatures were attributed to stimulated substrate availability, providing a favorable environment for soil microbes (Zhou et al., 2017). However, decreased soil moisture and pH provided unfavorable elements for the soil microbial community (Figure 2). In an alpine meadow, warming altered microbial community composition due to decreased pH and vegetation coverage causing an unfavorable environment for microbial community (Yu et al., 2019). Consequently, these antagonistic abiotic and biotic factors may have offset each other, resulting in the absence of a warming effect on soil microbial biomass and community composition (Figures 3 and 4). These results suggest an adaptation of the soil microbial community to short‐term warming in the studied semiarid grassland ecosystem.

A review study on terrestrial ecosystems reported that a shift in soil microbial community composition occurred after an average of three years of warming (Allison & Martiny, 2008). In some cases, even more than a decade may be required to detect significant warming responses of the microbial community composition (Rinnan et al., 2007). However, Radujkovic et al., (2018) reported that long‐term (>50 years) and even long‐term (5–7 years) soil warming did not induce the shifts of soil microbial community composition, but significant shifts for bacteria and fungi were observed starting from +9°C in the long term and +7°C/+3°C in the short term in the subarctic grasslands. Thus, long‐term or higher temperature increase experiments are also necessary to detect whether there are significant responses of soil microbial biomass and community composition to warming in grasslands. Such long‐term experiments are planned at the site.

Our hypothesis that warming would decrease microbial respiration was confirmed. This result is consistent with studies in the semiarid alpine steppes (Liu et al., 2009; Zhao et al., 2019; Zhou et al., 2013). Previous studies have shown that the warming response of microbial respiration depends on various abiotic and biotic determinants, such as substrate N availability, pH, soil temperature and moisture, microbial C use efficiency, and plant production (Bradford et al., 2010; Chang et al., 2014; Hicks Pries et al., 2015; Li et al., 2013; Moyano et al., 2007; Peng et al., 2015; Ye et al., 2019, 2020; Zhou et al., 2007). Wan et al. (2007) reported that warming increased aboveground biomass, subsequently causing soil available N increasing in a tallgrass prairie (Wan et al., 2005), which had a positive effect on soil microbial respiration (Hadas et al., 2004). However, too high inorganic N may induce N toxicity (decreased osmotic potential or pH), which can inhibit soil microbial respiration (Treseder, 2008). The northwest area of China received a high N deposition rate with 2.21 g m^−2^ yr^−1^ (Liu et al., 2011), where soil microbes may experience N toxicity in a very high inorganic soil induced by warming (Figure 2h). Zeng et al. (2018) also reported that the potential positive effect of soil inorganic N on soil microbial respiration was suppressed by the negative effect from decreased pH in temperate arid grasslands (Zeng et al., 2018).

In addition, in warming treatments, the positive effect of increased temperature on microbial respiration could be suppressed by decreased soil water content or water stress induced by warming (Chang et al., 2014; Y. Liu et al., 2016). Many field experiments found that warming reduced soil moisture (Hicks Pries et al., 2015; Tao et al., 2013; Wan et al., 2007; X. Wang et al., 2014). Water is the main limiting factor in semiarid or arid ecosystems (Walter et al., 2011), and reduced soil moisture can decrease microbial metabolic rate (W. Liu et al., 2009). A former study in the same site showed that around 2‐year warming (April 2015 to December 2016) decreased microbial respiration, but the overall driving mechanisms remained unclear (Fang et al., 2018). To evaluate whether the reduced soil water content or the other soil factors underlay the warming response of microbial respiration, SEM was used to provide a better and more systematic understanding.

The results of SEM suggested that the decreased soil moisture and pH, and increased AGB and inorganic N codetermined the negative warming response of soil microbial respiration (Figure 5). The decline in soil microbial respiration following warming was directly linked to changes in soil pH, and indirectly linked to changes in AGB, inorganic N, and soil moisture. On the one hand, increased AGB decreased soil moisture, which subsequently had a positive effect on pH, causing a negative effect on soil microbial respiration. On the other hand, negative effect of inorganic N on pH overrode the positive effect of AGB inducing an observably decreased pH, causing a negative warming effect on soil microbial respiration. These driving pathways demonstrated that warming responses of microbial respiration resulted from a combination of multidriving paths from abiotic and biotic factors.

In conclusion, we observed that warming did not change soil microbial community composition and biomass but decreased soil microbial respiration. The lack of warming responses of soil microbial community composition and biomass was due to their adaptation to the short‐term and limited (2°C) warming. On the other hand, the negative effects of inorganic N on pH overrode the positive effects of AGB, inducing an overall decrease in pH. This caused a negative effect of warming on soil microbial respiration. These driving pathways demonstrated that the warming responses of microbial respiration resulted collectively from multiple abiotic and biotic factors. Decrease in MMQ further supported our conclusion: Microbial community will decrease metabolic rate to adapt to more severe/harsh environments. These findings further revealed that in the short term the soil microbial community can resist to a warmer climate by decreasing their metabolic rate in the semiarid grasslands.

## CONFLICT OF INTEREST

None declared.

## AUTHOR CONTRIBUTION

**Chao Fang:** Conceptualization (equal); Data curation (lead); Formal analysis (lead); Funding acquisition (supporting); Investigation (lead); Methodology (lead); Project administration (supporting); Resources (equal); Supervision (equal); Validation (lead); Visualization (equal); Writing‐original draft (equal); Writing‐review & editing (equal). **Bin Wen Ke:** Data curation (supporting); Formal analysis (equal); Investigation (supporting); Methodology (supporting); Project administration (supporting); Resources (supporting); Validation (supporting); Visualization (equal); Writing‐original draft (equal); Writing‐review & editing (equal). **Matteo Campioli:** Formal analysis (equal); Investigation (supporting); Methodology (equal); Resources (equal); Supervision (equal); Validation (equal); Writing‐original draft (lead); Writing‐review & editing (lead). **Ying Jiu Pei:** Conceptualization (equal); Formal analysis (equal); Investigation (equal); Project administration (supporting); Resources (equal); Supervision (equal); Writing‐original draft (supporting); Writing‐review & editing (supporting). **Zi‐Qiang Yuan:** Formal analysis (equal); Investigation (equal); Supervision (equal); Validation (equal); Writing‐original draft (equal); Writing‐review & editing (equal). **Xin Song:** Formal analysis (equal); Investigation (equal); Supervision (equal); Validation (supporting); Writing‐original draft (supporting); Writing‐review & editing (supporting). **Sheng Jian Ye:** Conceptualization (equal); Data curation (equal); Formal analysis (equal); Funding acquisition (lead); Investigation (lead); Methodology (lead); Project administration (lead); Resources (equal); Supervision (equal); Validation (equal); Writing‐original draft (lead); Writing‐review & editing (lead). **FengMin Li:** Conceptualization (equal); Data curation (equal); Formal analysis (equal); Investigation (equal); Methodology (equal); Project administration (equal); Supervision (equal); Validation (equal); Writing‐original draft (lead); Writing‐review & editing (lead). **Ivan A. Janssens:** Formal analysis (lead); Investigation (supporting); Methodology (equal); Project administration (supporting); Resources (equal); Supervision (equal); Validation (equal); Writing‐original draft (lead); Writing‐review & editing (lead).

## Supporting information

Appendix S1

## Data Availability

An archive containing dataset and scripts to reproduce analyses can be downloaded at https://doi.org/10.5061/dryad.ffbg79cs9.
