# Supplementary material for: Unaltered soil microbial community composition, but decreased metabolic activity in a semiarid grassland after two years of passive experimental warming
Source: Ecol Evol. 2020 Oct 8;10(21):12327–40. doi: 10.1002/ece3.6862 (PMC7664004; doi:10.1002/ece3.6862)

Appendix A

Table A Linear mixed models between mean microbial respiration from June to September 2017 (Rh) and soil inorganic nitrogen (ION), pH, aboveground biomass (AGB) and soil water content (SWC). F, F values of the model; **p**, *p* values of the model; R^2^, R squared values of the model; AIC, Akaike information criterion of the models. Bold represents the best model with the lowest AIC value.

| Model | F | *p* | R^2^ | AIC |
| --- | --- | --- | --- | --- |
| Rh=-0.03ION+2.33 | 16.000 | 0.003 | 0.63 | -7.70 |
| Rh=1.35pH-9.51 | 17.070 | 0.003 | 0.64 | -8.14 |
| Rh=-0.002AGB+2.37 | 4.437 | 0.068 | 0.28 | -1.12 |
| Rh=0.087SWC+1.44 | 4.636 | 0.063 | 0.29 | -1.28 |
| Rh=-0.015ION+0.779pH-4.497 | 8.464 | 0.014 | 0.62 | -7.00 |
| Rh=-0.029ION-0.0004AGB+2.363 | 7.290 | 0.019 | 0.58 | -5.97 |
| Rh=-0.027ION+0.042SWC+1.977 | 9.729 | 0.010 | 0.66 | -8.01 |
| Rh=1.141pH-0.001AGB-7.593 | 9.927 | 0.009 | 0.66 | **-8.16** |
| Rh=1.21pH+0.019SWC-8.46 | 7.836 | 0.016 | 0.60 | -6.46 |
| Rh=-0.001AGB+0.0597SWC+1.838 | 3.343 | 0.095 | 0.34 | -1.42 |
| Rh=-0.0028ION+1.051pH-0.0009AGB-6.806 | 5.690 | 0.034 | 0.61 | -6.18 |
| Rh=-0.0217ION+0.268pH+0.0357SWC-0.319 | 5.649 | 0.035 | 0.61 | -6.13 |
| Rh=-0.026ION-0.0001AGB+0.0414SWC+1.995 | 5.574 | 0.036 | 0.60 | -6.04 |
| Rh=1.097pH-0.001AGB+0.007SWC-7.275 | 5.714 | 0.034 | 0.61 | -6.21 |
| Rh=-0.0112ION+0.656pH-0.0006AGB+0.0205SWC-3.545 | 3.665 | 0.093 | 0.54 | -4.40 |

**Fig. A** Conceptual structure equation modelling (SEM) with variables (boxes) and potential causal relationships (arrows) for soil microbial respiration. Rh: Microbial respiration; SWC: soil water content; pH: soil pH value; AGB: aboveground biomass; ION: inorganic nitrogen.


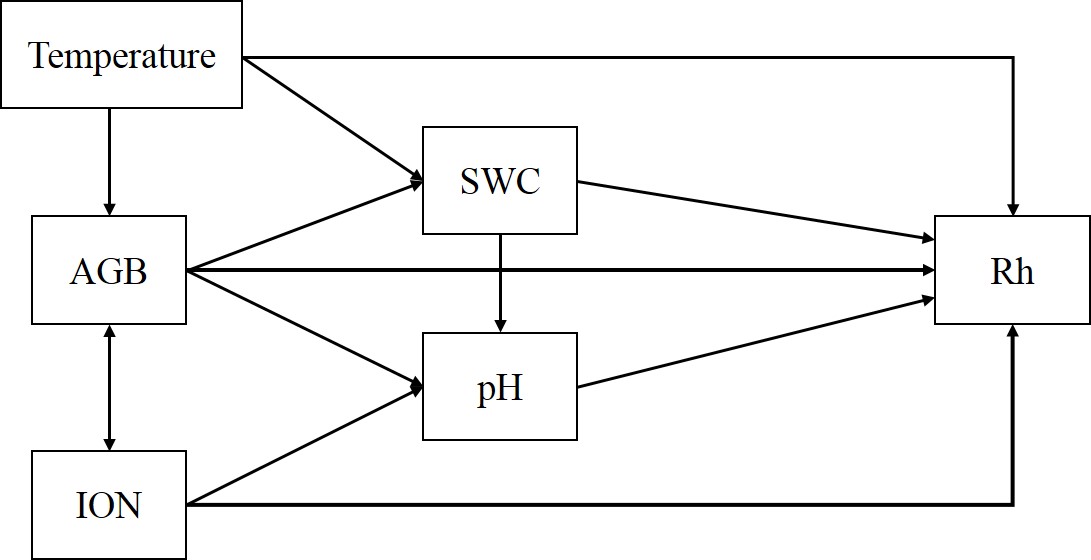

Supplement: Supplementary file 1 — Appendix S1 [file ECE3-10-12327-s001.docx]
